# Improved Location Estimation in Wireless Sensor Networks Using a Vector-Based Swarm Optimized Connected Dominating Set

**DOI:** 10.3390/s19020376

**Published:** 2019-01-17

**Authors:** Gulshan Kumar, Rahul Saha, Mritunjay Kumar Rai, Reji Thomas, Tai-Hoon Kim, Se-Jung Lim, Jai Sukh Paul Singh

**Affiliations:** 1 Department of Computer Science and Engineering, Lovely Professional University, Jalandhar-Delhi GT Road, Phagwara 144 441, India; rsahaaot@gmail.com; 2 Department of Electronics and Electrical Engineering, Lovely Professional University, Jalandhar-Delhi GT Road, Phagwara 144 441, India; raimritunjay@gmail.com (M.K.R.); khalsa128@gmail.com (J.S.P.S.); 3 Division of Research and Development, Lovely Professional University, Jalandhar-Delhi GT Road, Phagwara 144 441, India; rthomas.eyyalil@gmail.com; 4 Department of Convergence Security, Sungshin Women’s University, Seoul 02844, Korea; taihoonn@daum.net; 5 Department of Computer Engineering, Chonnam National University, 50 Daehak-ro, Yeosu 59626, Jeollanam-do, Korea; limsejung@korea.ac.kr

**Keywords:** localization, accuracy, load balance, swarm optimization, network lifetime

## Abstract

Location estimation in wireless sensor networks (WSNs) has received tremendous attention in recent times. Improved technology and efficient algorithms systematically empower WSNs with precise location identification. However, while algorithms are efficient in improving the location estimation error, the factor of the network lifetime has not been researched thoroughly. In addition, algorithms are not optimized in balancing the load among nodes, which reduces the overall network lifetime. In this paper, we have proposed an algorithm that balances the load of computation for location estimation among the anchor nodes. We have used vector-based swarm optimization on the connected dominating set (CDS), consisting of anchor nodes for that purpose. In this algorithm, major tasks are performed by the base station with a minimum number of messages exchanged by anchor nodes and unknown nodes. The simulation results showed that the proposed algorithm significantly improves the network lifetime and reduces the location estimation error. Furthermore, the proposed optimized CDS is capable of providing a global optimum solution with a minimum number of iterations.

## 1. Introduction

Wireless sensor networks (WSNs) are widely used in observing, agriculture, home, and defence environments that are dynamic. Emerging information technology and application requirements have shifted the paradigm of a static network scenario to dynamic environments of WSNs. The mobility of nodes (anchor and unknown) in the network makes it difficult to estimate the location of an unknown node. The accuracy of the location of nodes in such networks is of great importance and depends on location estimation and verification [1,2]. The major drawback of WSNs is the lifetime of the network, as the nodes have limited resources to power up. Compared to unknown nodes, anchor nodes are more privileged, with better power and computational resources. However, unknown nodes drain their resources faster to perform heavy computational tasks. Therefore, the network becomes partially unavailable until the exhausted nodes are replaced. However, the replacement process is not always feasible in the dynamic configuration settings of the applications. Moreover, WSNs use a virtual backbone to prolong the network lifetime [3,4,5]. Therefore, the localization process has to be efficient in the terms of minimizing resource utilization. Such efficient utilization of resources is achieved by optimization [6,7,8] and load balancing [9] on the virtual backbones.

In this paper, we introduce an approach by constructing a connected dominating set (CDS) based virtual backbone only with anchor nodes optimized in terms of load balance using a vector-based swarm optimization (VBSO) mechanism [10]. This optimized CDS is then used for location estimation in WSNs. The purpose of including CDS in the proposed algorithm is to use the anchor nodes properly and to increase the overall network lifetime. The remaining paper is organized as follows. Section 2 reviews the related work in this field. The network model for the algorithm is explained in Section 3. The proposed algorithm is shown in Section 4. The detailed process of the applied optimization is explained in Section 5, and the localization details are discussed in Section 6. The results are explained in Section 7. Finally, conclusions are drawn in Section 8.

## 2. Related Work

Researchers have recently considered various algorithms to improve the lifetime of nodes without compromising their location estimation accuracy. Localization techniques are broadly categorized into (i) direct and (ii) indirect approaches. The former is based on manual configuration and GPS-based techniques and the latter on range-based and range-free techniques [1]. Location identification of nodes with range-based approaches uses different methods, such as time difference of arrival (TDoA), angle of arrival (AoA), time of arrival (ToA), and a received signal strength indicator (RSSI). On the other hand, range-free approaches use region inclusion, hop count, and neighbor location identification methods that avoid a direct relationship between point-to-point distances and are, therefore, more economic. Some hybrid approaches, including RSS–AoA and RSS–ToA, have been discussed in References [11,12,13,14]. These hybrid approaches provide generality by applying data fusion and are also used for global synchronization with high accuracy but lag behind in computational cost, special hardware requirements, and energy consumption. Another hybrid localization algorithm is shown in Reference [15] that uses message passing and approximation of posterior distribution of targets. It also uses Bayesian distribution [16,17]. These techniques either use range or angle as their measurement parameter and geometrical interpretations (such as triangulation or trilateration) are calculated accordingly. This seems easier to apply in the absence of noise. Moreover, the problem with such a geometric-based calculation is the existence of a larger intersection area of hyperbolas rather than a point. Therefore, to improve the efficiency of the localization techniques in the presence of noise, the two measurements—range and angle—are integrated as hybrid approaches. The generic approaches of range-free methods were recently further modified for their economic value and are discussed hereafter. Energy-efficient load- balanced clustering (EELBC) is one of the range-free approaches for localization [18]. This algorithm uses a minimum heap of cluster heads (CHs) or gateway nodes and the nodes are assumed to be aware of the position through GPS. Additionally, network setup is performed in two phases, bootstrapping and clustering. In the clustering phase, the sink executes the clustering algorithm depending on the minimum heap algorithm. The extra cost of GPS makes this algorithm less attractive.

Another variant of the load-balanced cluster-based algorithm creates clusters based on distances and the distribution density of the nodes [19]. Here, node distribution follows Poisson’s process and has fixed locations and, hence, mobility is not considered. This algorithm lacks load balancing factors. In another range-free approach, three NP-hard problems: the min–max degree maximal independent set (MDMIS), the load-balanced virtual backbone (LBVB), and the min–max valid-degree nonbackbone node allocation (MVBA) were considered [20]. An additional approximation algorithm was also proposed in the method which uses the linear relaxing and random rounding techniques. Genetic-based approaches have been discussed in References [21,22,23,24]. One variant of the genetic algorithm using a simplex method is shown in Reference [25]. This simplex method is used to optimize the error minimization problem in widely used DV-hop for searching a local optima. The use of the multiobjective genetic algorithm to create a load-balanced virtual backbone is shown in Reference [26]. The mobility of the network increases the iterations of the algorithm to get a local optimum solution, making the overall approach disadvantageous. The identification of balanced nodes in wireless sensor networks for data aggregation has also been shown with a probabilistic network model for a data aggregation tree [27]. Moreover, an algorithm for the identification of a load-balanced maximal dominator node Set (LBMDS) and connector nodes for LBMDS have been developed. An expected allocation probability algorithm (EAP) to solve parent node assignment (PNA) has been proposed. A novel distributed tracking process of the sensor nodes has been introduced [28]. In this approach, a local node using a fuzzy system provides a partial solution, with a centralized algorithm that merges all the partial solutions. The centralized algorithm is based on the calculation of the centroid of the partial solutions. The deviation of the partial solutions increases the complexity of the algorithm, which is an identified concern in this approach.

The solution for swarm configuration-based localization uses the min–max method and swarm optimization [29]. Swarm uses a sequential Monte Carlo localization method to consider mobility and does not require additional hardware. As it can work with seed movement, the mobility factor lowers the cost of implementation without compromising accuracy. We have considered this approach as a candidate, as well for comparison. An ant colony-based optimization approach is described in the literature [30]. A distributed method for parametric regression in a clustered WSN using particle swarm optimization was also introduced recently with multiobjective optimization (MO) [31]. Moreover, the same as the vector-evaluated particle swarm optimization method, it considers the MO problem in two phases; the algorithm obtains a set of candidate network regressors and computes the final model using a weighted averaging rule. However, this parametric regression is not suitable for real time-based scenarios with the mobility effect. A novel optimized localization method using glowworm swarm optimization (GSOL) has been introduced to overcome this drawback [32]. The fitness function of this optimization process follows the deviation or error calculation with the Euclidean distance aspect. After the multi-iterations, all the mobile nodes within the network concentrates around one or multiple nodes providing local optimal location create the global optimal location. We have considered this algorithm also as another candidate for comparison due to its claimed efficiency. Some other techniques for the optimization and load balancing the virtual backbone include: Neural networks [33], the machine learning approach [34], and the differential evolution approach [35].

The aforementioned short survey on the range-free optimization algorithms in WSNs has some drawbacks: (i) The algorithms are not load-balanced and, therefore, partial exhaustion of the network exists; (ii) the redundancy of the clustering nodes makes the process complex; (iii) heavy functioning load on some nodes lowers the network lifetime; and (iv) validation for the overall mobile (unknown and anchor: Both are mobile) environment is not ensured. The present work aims to provide a solution for the abovementioned deficiencies by developing an approach of localization using a VBSO-based CDS construction.

## 3. Network Scenario

The present research work considers that the *n* unknown nodes are located within a plane of M×M area with true locations defined by $u_{i}=\left(x_{i},y_{i}\right)$ and estimated locations are defined by $\bar{u_{i}}=\left(\bar{x_{i}},\bar{y_{i}}\right)$, ∀i=1,2,..,n. We have also considered *m* anchor nodes with known locations defined by $a_{j}=\left(x_{j},y_{j}\right),\forall j=1,2,..,m$.

The transmission area is considered to be a circle. The centre of the circle consists of the corresponding node itself. The number of unknown nodes connected to an anchor node is variable at different time intervals and, therefore, we have calculated that the expected number of connectivity for each anchor node in the network can be given as:

(1) $$\zeta =\frac{n}{M^{2}}\pi r^{2}.$$

The network of *n* unknown nodes and *m* anchor nodes along with their connectivity is considered as a graph G(V,E). $G^{\prime }\left(V^{{}^{\prime }},E^{\prime }\right)$⊆G(V,E), where G(V,E) is the original sensor network scenario and $G^{{}^{\prime }}(V^{{}^{\prime }},E^{{}^{\prime }})$ consists only of the optimized backbone anchor nodes considered as optimized CDS and its links to the unknown nodes $u_{1},u_{2},\ldots ,u_{n}\in U$, the set of unknown nodes, such that $\forall u_{i},a_{j}\in V^{{}^{\prime }}${i=1,2,..,n and j=1,2,..,m}. All the unknown nodes in the set *U* are the one-hop neighbors of any anchor node present in the CDS such that D⊆A, the set of anchor nodes $\left\{a_{1},a_{2},..,a_{m}\right\}$, $V^{{}^{\prime }}\subseteq V$ and $V^{{}^{\prime }}=D\cup U$, where *D* is the dominating set.

The proposed network model is mobile in nature for both the anchor nodes and unknown nodes. Relative mobility is required to be calculated to check the mobility direction and the relative distances. The relative mobility between an unknown node $u_{i}$ with respect to anchor node $a_{j}$ at a given time *t* is given by:

(2) $$RM_{t}^{a,u}=d_{a,u_{t}}-d_{a,u_{t-1}},$$

where $d_{a,u_{t}}$ = $\frac{k}{\sqrt{P_{r}}}$ and $P_{r}$ is the power received by the receiving antenna and is calculated by the Friis space model [36]. *k* is constant here. $d_{a,u_{t}}$ is used to calculate the distance and closeness between two nodes. $RM_{t}^{a,u}$ is positive if node *u* is moving away from *a* and negative if *u* is coming closer to *a*. This relative mobility effects the CDS generation and further the related vectors. This relative mobility is used to generate the updated distance matrices for location estimation.

## 4. Vector-Based Swarm Optimized CDS

Our proposed algorithm considers the following assumptions:

- The anchor nodes are preinstalled with their own location, also known to the base station (BS).
- The base station can control all the anchor and unknown nodes. Multiple base stations are allowed, depending on the range of communication.
- Unknown nodes and anchor nodes have random mobility.
- According to the privilege concern, BS is the most privileged in terms of storage and resource. Anchor nodes are more privileged, compared to unknown nodes. Therefore, the maximum computing tasks are controlled by BS; anchor nodes and unknown nodes are kept only with a minimum message exchange process. One BS broadcasts the aggregated information to other BS.

The proposed method starts with broadcasting by anchor nodes which have more privileges than the resource constrained unknown nodes. The anchor nodes broadcast the neighbor discovery packet (NDP) as per the following format.

NDP:{A_addr,Leash_C=1,Seq,Accept_S=0},

where A_addr is the source address of the anchor node, Leash_C is set to 1 to bound the packet with a limited transmission of one-hop nodes, Seq is the sequence number of the NDP to avoid void routing and stale of packets, and Accept_S is the status of the acceptance of the NDP by an unknown node set at 0. It may happen that in this process of broadcast, another anchor node receives the NDP, in which case, the receiving anchor node will simply discard the NDP. Once this NDP is received by the unknown nodes who are in the one-hop neighborhood of the sender, the unknown nodes reply with a reply neighbor set (RPNS) by decrementing the leash value by 1 and changing the acceptance status to 1. The RPNS is composed of the following components.

RPNS:{SrcAddr,Leash_C=0,Seq,Accept_S=1,U_addr}|U_addristheaddressofunknownnode.

Upon receiving the RPNS, the anchor nodes enlist the U_addr as its one-hop neighbor in a neighbor set list (NSL) and send it to the base station (BS) with cryptographic services so that the list cannot be manipulated by any third party attack or compromised insider node.

$$Anchor\to BS:NSL=\{A\_addr|U\_addr_{1},U\_addr_{2},\ldots \}$$

After receiving all the NSLs from the anchor nodes, BS calculates the maximum degree anchor node and enlists the anchor node in an empty dominating set D. This operation iterates until at least three anchor nodes are in the dominating set and all the unknown nodes are a one-hop neighbor of at least one of the anchor nodes in the set of anchor nodes A. The process outputs a connected dominating set (CDS), but the problem is the anchor nodes are unbalanced in terms of number connections with unknown nodes. To solve this problem, swarm optimization is performed on the CDS. BS creates binary vector $V_{j}$ for each of the anchor nodes present in the CDS and vector-based swarm optimization is applied for the best solution to achieve an optimized CDS for the backbone. All these *n*-bit binary vectors (as number of unknown nodes is *n*) create the initial population of swarm. Fitness value is calculated on each of these vectors and direct and indirect cooperation is calculated. After mutation on the initial population of vectors, boundary values are checked and selection starts again for the next iteration. If the convergence condition is met successfully, we get the output of the best-fit solution for our purpose. Once we get the optimized dominating set, trilateration is performed to get the location of unknown nodes. The algorithm is summarized in Algorithm 1.

**Algorithm 1** Proposed Localization Algorithm
1:**procedure** Anchor nodes $a_{j}$ and unknown nodes $u_{i}$2:    Initialize $\left\{A\right\}=\left\{a_{1},a_{2},\ldots ,a_{m}\right\}$ and $\left\{U\right\}=\left\{u_{1},u_{2},\ldots \ldots ,u_{n}\right\}$ and dominating set D=∅3:    **for** Broadcast by anchor node $a_{j}$ **do**4:        Send NDP:{A_addr,Leash_C=1,Seq,Accept_S=0}5:    **end for**6:    **for** On receiving, node $u_{i}$ computes **do**7:        Leash_C = Leash_C - 1;8:        Accept_S = 1;9:        Send RPNS:{A_addr,Leash_C,Seq,Accept_S=1,U_addr}10:    **end for**11:    **if** Leash_C==0&&Accept_S==1 **then**12:        Create NSL13:        $a_{j}\to BS:NSL=\left\{A\_addr|U\_addr_{1},U\_addr_{2},\ldots \ldots \right\}$14:    **else**15:        exit16:    **end if**17:    BS performs:18:    **while** True **do**19:        **if** $\forall \;u_{i}\;\in \;(\exists \;N\;\left(a_{j}\right)\;\&\&\;|D|\;\geq 3)$ **then**20:           Break while loop;21:        **else**22:           Calculate the maximum degree anchor nodes $a_{max}$23:           $D=D\;\cup \;a_{max}$24:        **end if**25:    **end while**26:    $m^{\prime }=\left|D\right|$27:    **for** k = 1 to m’ **do**28:        BS creates binary vector $\vee_{j},\forall_{j}=1,2,\ldots ,m^{\prime }$29:        Apply VBSO algorithm30:        Apply Trilateration31:    **end for**32:
**end procedure**



## 5. Optimization Process with VBSO

To optimize the obtained CDS, we have applied vector-based swarm optimization (VBSO) on the obtained CDS. The solution for optimizing the CDS exhibits the features of the maximization problem. To solve this problem, we considered the connections of anchor nodes in the CDS with their corresponding one-hop neighboring unknown nodes as *n*-dimensional vector $V_{j}$. The proposed method provides a global maximization solution, which is formulated as a pair $\left(V_{j},f\right)$, where $V_{j}\subseteq R^{n}$ is a bounded set on $R^{n}$ and $f:V_{j}\to R$ is *n* dimensional real valued function. The domain $R^{n}$ of function *f* is considered the search space. We have to find a point $\hat{x}\in V_{j}$ so that $f(\hat{x})$ is a global maximum on $V_{k}$. Thus, each element *x* of $R^{n}$ is considered a candidate solution in the defined search space with $\hat{x}$ being optimal such that $f\left(\hat{x}\right)\leq f\left(x\right)$. The optimization process for CDS with VBSO algorithm has been summarized in Algorithm 2.

### 5.1. Initialization of Vectors

Each unknown node in the network is allocated to one of the anchor nodes in the CDS. Each anchor node $a_{j}$ and its connections with one-hop neighboring unknown nodes $u_{i}$ is represented by an n-dimensional binary vector $V_{j},\forall j=1,2,..,m^{{}^{\prime }}$, where $m^{{}^{\prime }}$ is the number of anchor nodes in CDS and $m^{{}^{\prime }}\subseteq m$.

**Figure 1:**
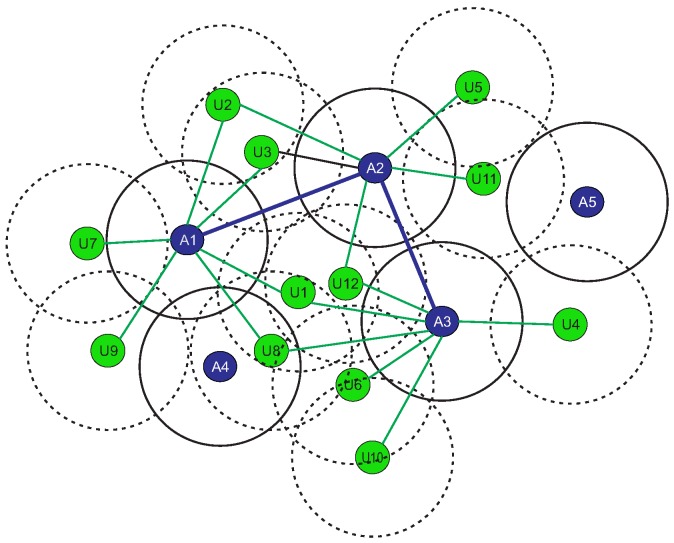
Example scenario.

**Figure 2:**
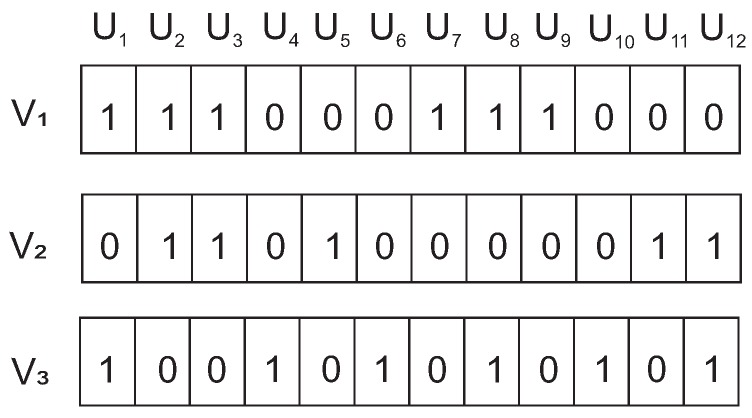
Vector representation for connected dominating set (CDS) shown in Figure 1.

Let us consider the scenario shown in Figure 1. The scenario consists of five anchor nodes and twelve unknown nodes. The blue circles represent anchor nodes, green circle nodes represent unknown nodes, blue solid lines represent a CDS and green solid lines represent the connection between an anchor node of CDS and unknown node in the network. As per our algorithm, we require at least three anchor nodes in the CDS to calculate a location with the trilateration process. Therefore, anchor nodes 1, 2, and 3 create the required CDS for the purpose for which the vector representation (V1, V2, and V3) is shown in Figure 2. These vectors are considered parent vectors for the VBSO algorithm. The vector representation for each anchor node is formulated as:

(3) $$V_{j_{i}}=\left\{\begin{array}{c} 1,\;\;if\;anchor\;a_{j}\;is\;one\;hop\;neighbour\;of\;u_{i}\\ 0,\;\;else \end{array}\right.\;\;\forall \;i=1,2,..,n\;and\;\forall \;j=1,2,..,m^{{}^{\prime }}$$

### 5.2. Calculate the Fitness Vector

The formulation of the fitness function is a critical and important task in the optimization process, as it helps for fast convergence with the optimum solution. To formulate the fitness function, we have used two parameters, which are also considered in our previous work [37].

Dominating set θ value for the connected dominating set D given as: $|CDS_{\theta }|={\left(\sum_{j=1}^{m^{{}^{\prime }}}{\left|deg_{j}-d\bar{e}g\right|}^{\theta }\right)}^{\frac{1}{\theta }}$, where $deg_{j}$ is the degree of each dominator anchor node in the set *D* and $d\bar{e}g$ is the mean of all the degrees of the dominator in the set *D*. The calculation of allocation scheme value: |$Al_{\theta }|={\left(\sum_{j=1}^{m^{{}^{\prime }}}{\left|deg_{j}^{{}^{\prime }}-\zeta \right|}^{\theta }\right)}^{\frac{1}{\theta }}$ considers that there are $m^{{}^{\prime }}$ disjointed sets for $m^{{}^{\prime }}$ anchor nodes present in the set *D*, such that $N\left(a_{j}\right)\cap N\left(a_{k}\right)=\emptyset$ and $\forall u_{i}\in N\left(a_{j}\right),1\leq j\leq m^{{}^{\prime }}$ so that $\left(u_{i},a_{i}\right)\in E^{{}^{\prime }}$. N() here represents the neighborhood. $deg_{i}^{{}^{\prime }}$ is the valid degree of an anchor node $a_{j}$, i.e., the number of unknown nodes already allocated to the anchor node $a_{j}$.

The objective of the problem defined in this paper is to identify an optimized CDS with minimum $D_{\theta }$ and $Al_{\theta }$ values. Therefore, the fitness function can be given as:

(4) $$\left\{\begin{array}{c} f\left(V_{j}\right)=\frac{n-|D|}{W_{1}|CDS_{\theta }|+W_{2}\left|Al_{\theta }\right|}\;\\ W_{1}+W_{2}=1,such\;that\;0<W_{1},W_{2}<1 \end{array}\right.$$

The linear combination of the two metrics $CDS_{\theta }$ and $Al_{\theta }$ is made to adjust the bias factors $W_{1}$ and $W_{2}$, according to the network environment. The above equation emphasizes that to maximize the fitness function for achieving optimum value, the denominator needs to be minimized and the numerator needs to be maximized so that the overall fitness function will be maximized accordingly. The experimented value of θ = 2, as it is analogous for an electro static field.

### 5.3. Reproduction in VBSO

The reproduction method in the VBSO algorithm primarily depends on creating cooperation vectors. Multiple vectors in the search space are combined with vector operations to achieve the cooperation vector.

**Figure 3:**
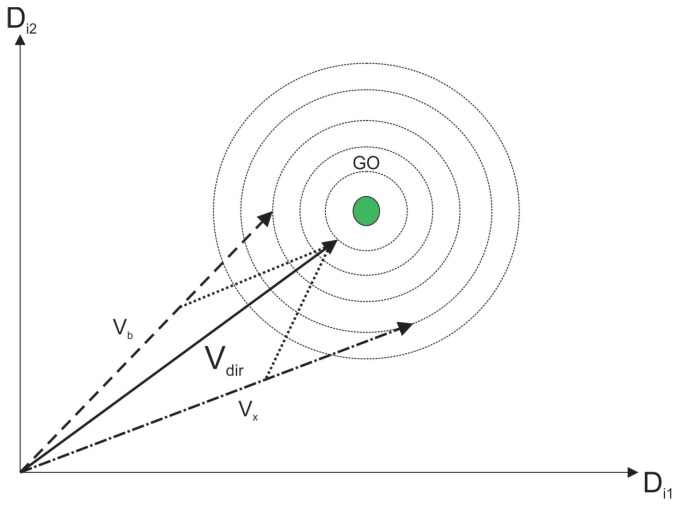
Global optima with $V_{x}$ and $V_{b}$.

**Figure 4:**
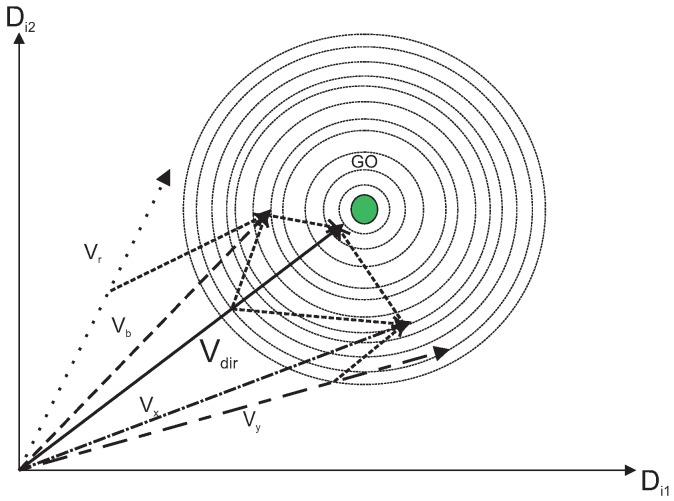
Direct cooperation vector.

The direct cooperation vector and differential cooperation vectors are used to create a cooperation vector. The direct cooperation vector at the kth iteration is given by:

(5) $$V_{dir}=w_{1}\cdot V_{x}+w_{2}\cdot V_{y}+w_{3}\cdot V_{z}+w_{4}\cdot V_{b}+w_{5}\cdot V_{r},$$

where $V_{x}$,$V_{y}$,$V_{z}$ belongs to the initial population vectors, $V_{b}$ is the best vector in the neighborhood, and $V_{r}$ is the random vector and $w_{1}$ + $w_{2}$ + $w_{3}$ + $w_{4}$ + $w_{5}$ =1. These values are called cooperative weighting coefficients (CWC). Figure 3 represents a direct cooperation vector with $V_{x}$ and $V_{b}$ which actually outputs the global optimum (GO). Following the same line, Figure 4 represents another direct cooperation vector with $V_{x}$, $V_{y}$, $V_{b}$m and $V_{r}$.

For the scenario of more than three initial population vectors, the selection of three among them is another crucial part which depends on higher fitness values and with a higher probability of selection. The probability is therefore given as:

(6) $$P\left(V_{j}\right)=\frac{f(V_{j})}{\sum_{j=1}^{m^{{}^{\prime }}}f\left(V_{j}\right)}.$$

**Figure 5:**
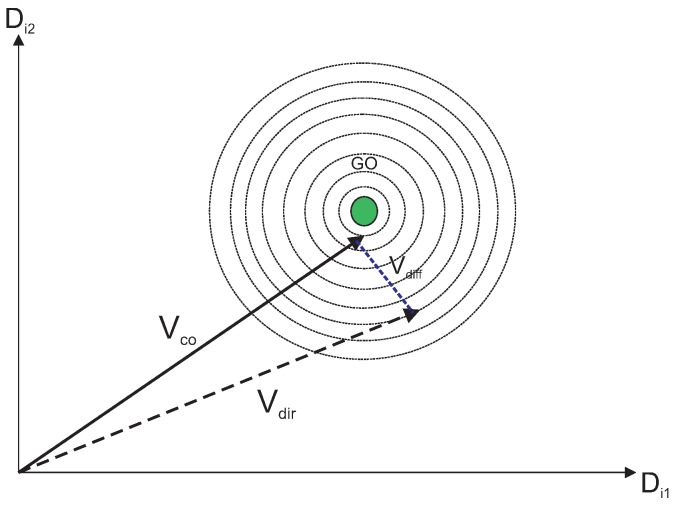
Differential cooperation vector creation.

The differential cooperation vector signifies the search around direct cooperation vector. It helps the algorithm to pass local optimums and converge to global one, as shown in Figure 5. Direct cooperation vectors provide a local solution, whereas the differential cooperation vector uses these direct vectors to get the global optimal solution, which is as good or better than all the other feasible solutions. To obtain the unknown global optimum, we have followed *k* iterations, in which if two simultaneous iterations have output the same values of local optima, we have considered it the global optimum. The differential cooperation vector ($V_{diff}$) of the population is calculated by considering proper portions of differential vectors of a current solution, for example $V_{c}$, given as:

(7) $$V_{diff}=w_{6}\cdot \left(V_{x}-V_{c}\right)+w_{7}\cdot \left(V_{y}-V_{c}\right)+w_{8}\cdot \left(V_{z}-V_{c}\right)+w_{9}\cdot \left(V_{b}-V_{c}\right)+w_{10}\cdot \left(V_{r}-V_{c}\right)$$

(8) $$where\;w_{6}+w_{7}+w_{8}+w_{9}+w_{10}=1$$

**Figure 6:**
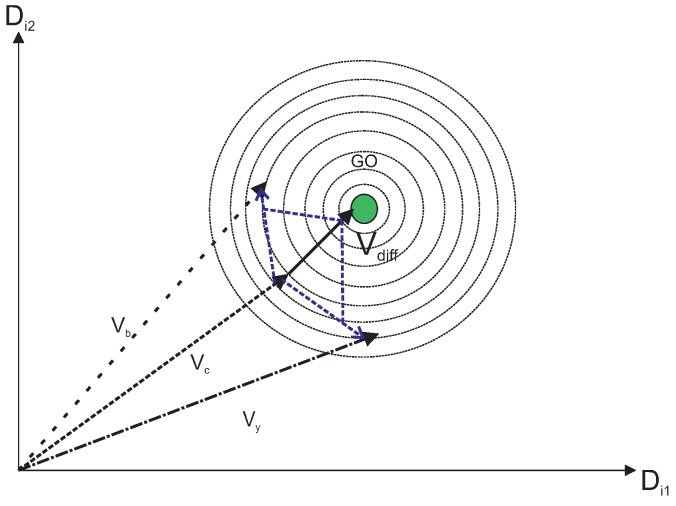
Generation of $V_{diff}$ with initial population vectors.

Figure 6 shows the generation of the cooperation vector obtained from direct and differential cooperation vectors, which can be given as:

(9) $$V_{co}=V_{diff}+V_{dir}.$$

**Figure 7:**
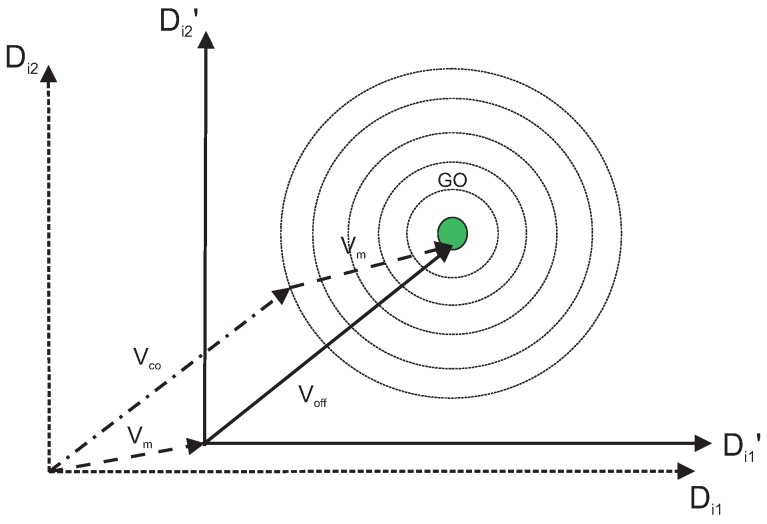
Candidate offspring generation with mutation vector.

In all Figure 3, Figure 4, Figure 5, Figure 6 and Figure 7, the contour lines represent the cost function that depends on the fitness value of the vectors.

### 5.4. Mutation

To increase the diversity of the population, mutation is applied to the initial population vectors. A number of mutation operators have been suggested in Reference [38,39,40]. Applying direct and differential cooperation implicitly provide mutation in the process. In addition, a second mutation is also applied to transfer the search space origin to a point far enough from the current origin. A random mutation probability $p_{m}$ is selected. A random number r is generated and then compared with $p_{m}$. The second mutation is applied if $rand\;>\;p_{m}$ and rand is in (0,1). In the mutation process, the random bits in a vector $V_{k}$ are flipped to get the mutation vector $V_{m}$. The candidate offspring vector $V_{off}$ is obtained by the following equation, and also shown in Figure 7:

(10) $$V_{off}=V_{m}+V_{co}.$$

### 5.5. Boundary Check

After the mutation, new offspring vectors are checked for the boundary condition so that all the offsprings must belong to the search space. An invalid offspring can lead to ab uncoordinated and invalidated search and the convergence will become a delay. Different methods are shown in References [41,42]. We have used the boundary check with a fitness function. If the fitness function of a vector in the current iteration is smaller than the fitness function of the best vector in the previous iteration, then replace the current unfit vector with its parent vector. The formulation of this process is given as:

(11) $$if\;f{\left(V_{j}\right)}_{i}<f{\left(V_{best}\right)}_{i-1}:f{\left(V_{best}\right)}_{i}=max(f{\left(V_{best}\right)}_{i-1},\;replace\;current\;vector\;with\;parent$$

**Figure 8:**
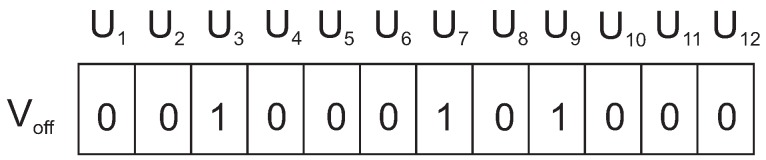
Offspring of the parent vectors shown in Figure 2.

For example, considering the scenario of Figure 2, the fitness value of the best solution is 5.89 (considering r=2m and θ=2 (analogy to electro static field). After reproduction and mutation, we have got the offspring vector as in Figure 8.

The candidate offspring has a fitness value of 0.278, which is less than the previous best solution of 5.89. Therefore, we need to replace this candidate with one of the parents of the previous iteration by calculating the hamming distance. This vector $V_{off}$ has a hamming distance of 3, 6, and 9 from the initial parent vectors $V_{1}$, $V_{2}$, and $V_{3}$, respectively. Thus, this candidate offspring will be replaced by vector $V_{1}$ that corresponds to $A_{1}$. This process of retaining the parent of best solution helps to converge faster to the global optimum.

### 5.6. Selection of Offsprings

**Figure 9:**
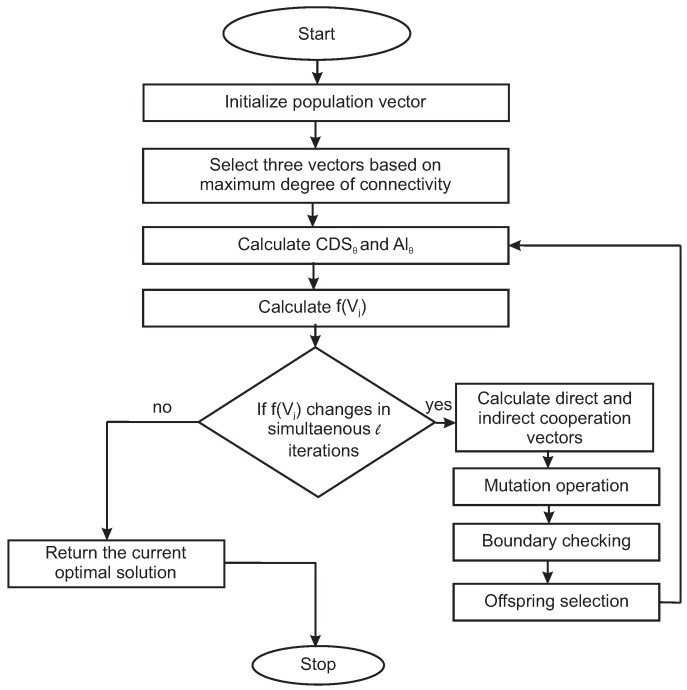
Flowchart of convergence process.

The identification of the next iteration vectors requires a selection process for suitable offspring vectors. Various methods of selection have been discussed in References [38,43,44]. In our proposed approach, all the valid candidate offsprings are directly transferred to the next iteration. The overall process of the vector-based optimization approach used in our purpose has been summarized in Algorithm 2. The convergence process of the optimization method is depicted in the form of a flowchart in Figure 9.

**Algorithm 2** VBSO Algorithm on CDS
1:**procedure** vector $\vee_{j},\;\forall ,\;j=1,2,...,m^{\prime }$2:    **while** $f(V_{j})$ does not change successive *l* iterations **do**3:        Calculate $|CDS_{\theta }|=(\sum_{j=1}^{m^{\prime }}|deg_{j}-\bar{deg}{{|}^{\theta })}^{\frac{1}{\theta }}$4:        Calculate expected allocated neighbour values as: $\zeta =\frac{n}{M^{2}}\pi r^{2}$5:        Calculate the allocation value given as: $|Al_{\theta }|=(\sum_{j=1}^{m^{\prime }}|deg_{j}^{\prime }-\zeta {{|}^{\theta })}^{\frac{1}{\theta }}$6:        Calculate $f\left(V_{j}\right)=\frac{n-|D|}{W_{1}|CDS_{\theta }|+W_{2}\left|Al_{\theta }\right|}$7:        **if** $f(V_{j})$ does not change in simultaneously *l* iterations **then**8:           goto Step 159:        **else**10:           Calculate direct and indirect cooperation vectors11:           Apply mutation12:           Check boundary condition13:           Selection of offsprings for next iteration14:           goto 615:           return the current optimal solution16:        **end if**17:    **end while**18:
**end procedure**



## 6. Localization of Unknown Nodes

Once the optimized CDS is achieved, we can apply any trilateration method to estimate the location of an unknown node $u_{i}$. The estimated distance ${\bar{d}}_{j}$ from an unknown node $u_{i}$ to each dominator anchor node in optimized CDS is given by the respective Euclidean distances between them, as shown below.

(12) $${{\bar{d}}_{j}}^{2}={\left(x_{j}-\bar{x}\right)}^{2}+{\left(y_{j}-\bar{y}\right)}^{2}|\;\forall \;j=1,2,..,m^{{}^{\prime }},$$

where $\left(\bar{x},\bar{y}\right)$ is the estimated location of an unknown node of $u_{i}$.

The calculated distance with relative mobility becomes:

(13) $$d_{j}={\bar{d}}_{j}+RM_{t}^{a,u}.$$

The above equation for $j=1,2,..,m^{{}^{\prime }}$ can be transformed into the AX=b form, where:

(14a) $$\begin{array}{c} A=\left|\begin{array}{cc} \left(x_{1}-x_{m^{{}^{\prime }}}\right) & \left(y_{1}-y_{m^{{}^{\prime }}}\right)\\ . & .\\ . & .\\ \left(x_{m^{{}^{\prime }}-1}-x_{m^{{}^{\prime }}}\right) & \left(y_{m^{{}^{\prime }}-1}-y_{m^{{}^{\prime }}}\right) \end{array}\right| \end{array}$$

(14b) $$\begin{array}{c} X=\left|\begin{array}{c} \bar{x}\\ \bar{y} \end{array}\right| \end{array}$$

(14c) $$\begin{array}{c} b=\frac{1}{2}\left|\begin{array}{cc} d_{1}^{2}-d_{m^{{}^{\prime }}}^{2}+d_{m^{{}^{\prime }}u}^{2}-d_{1u}^{2}\\ d_{2}^{2}-d_{m^{{}^{\prime }}}^{2}+d_{m^{{}^{\prime }}u}^{2}-d_{2u}^{2}\\ . & .\\ . & .\\ d_{m^{{}^{\prime }}-1}^{2}-d_{m^{{}^{\prime }}}^{2}+d_{m^{{}^{\prime }}u}^{2}-d_{(m^{{}^{\prime }}-1)u}^{2} \end{array}\right| \end{array}$$

Equation AX=b is solvable by the standards of the least-square [45] solution to provide the value of *x* given by:

(15) $$\bar{x}={\left(A^{T}A\right)}^{-1}A^{T}b.$$

The problems identified in the existing algorithms as stated in the section of related work have been addressed by our proposed solution and summarized in Table 1.

## 7. Result and Analysis

The proposed algorithm has been simulated in NS-2 with the following parameters enlisted in the Table 2.

The performance of the algorithm has been analyzed in the terms of localization error and network lifetime and number of iterations used to provide the global optimum solution. The location estimation error is defined as a squared error of the estimation and is given by:

(16) $$e_{i}=\sum_{j=1}^{m^{{}^{\prime }}}{\left(d_{j}-\bar{d_{j}}\right)}^{2},for\;j=1,2,..,m^{{}^{\prime }},$$

where $\bar{d_{j}}$ is the estimated distance and $d_{j}$ is the true distance between unknown node $u_{i}$ and anchor node $a_{j}$. The average error is given as:

(17) $$e_{avg}=\frac{\sum_{j=1}^{m^{{}^{\prime }}}e_{i}}{m^{{}^{\prime }}}$$

The statistical values of localization errors for our different simulation environment are shown in Table 3. Results are shown for the variable number of anchor nodes ranging from 10 to 40, with unknown nodes ranging from 100 to 200. The table also represents the number of anchor nodes used in the VBSO-optimized CDS, by which the given number of unknown nodes has been localized. The result signifies the fact that only 50% to 57% of all the anchor nodes are used. This means that the unused anchor nodes can save their energy for future tasks, which actually elongates the network lifetime.

**Figure 10:**
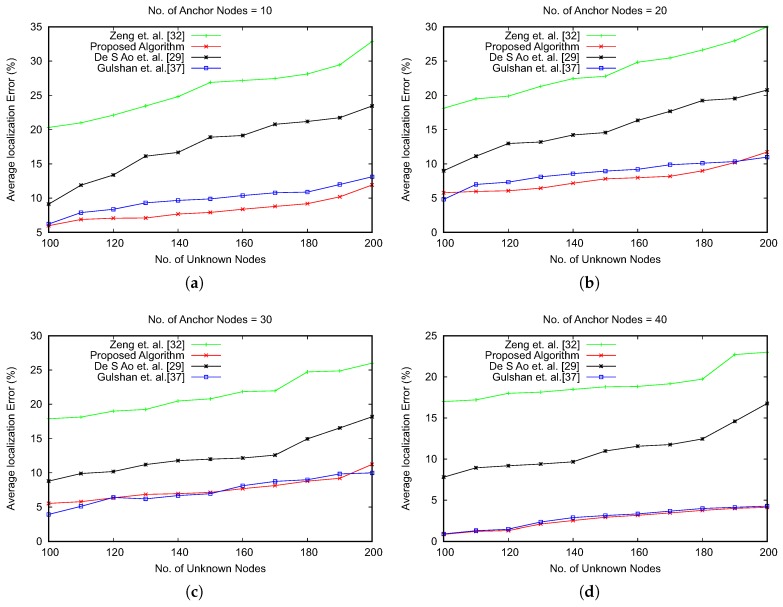
Average localization error comparison.

**Figure 11:**
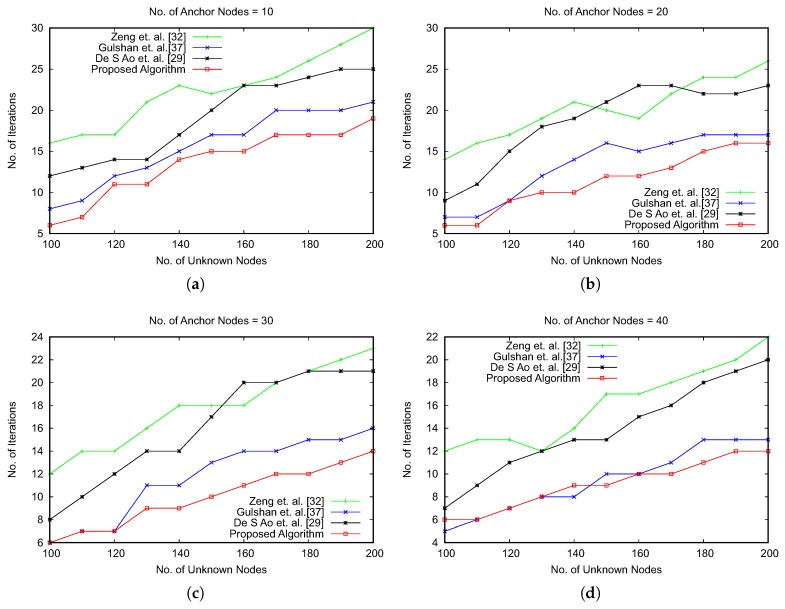
Comparison of iteration numbers with varying anchor nodes and unknown nodes.

The results of the average localization error have also been compared with three recent optimization-based approaches, as shown in References [29,32,37]. The comparison results are shown in Figure 10. The results emphasize that our proposed approach reduces the average error for localization of unknown nodes. We have observed that with the increasing number of anchor nodes, the proposed algorithm and the work in Reference [37] have a marginal difference of average localization error. However, the advantage of the proposed algorithm lies with the number of iterations for obtaining the global optimum, as shown in Figure 11. The optimization algorithms consider the number of iterations in searching the global optima as an important performance metric. We have compared the number of iterations executed by varying the number of anchor nodes and unknown nodes. Figure 11 shows the results of comparison, which signify that our proposed algorithm is faster in providing the global optimum value with a lower number of iterations of the optimization process.

The objective of the proposed algorithm is to elongate the network lifetime. Therefore, we have also evaluated the performance of the proposed algorithm in terms of residual energy. The proposed algorithm uses a linear energy model. The overall residual energy of the proposed optimized CDS depends on the constituents, i.e., the dominator anchor nodes. We have considered a timeline, say *t1* to *t2*, for this energy consumption measurement. Residual energy in time *t* is defined by omitting consumed energy in ▵t from the initial battery power in t−▵t. Thus, the energy consumption is determined in ▵t. Therefore, the residual energy equation is given as:

(18) $$E_{res}=\frac{\sum_{j=1}^{\left|D\right|}E_{init}{\left(t-▵t\right)}_{j}-E_{consmp}{\left(t-▵t\right)}_{j}}{\left|D\right|}$$

(19) $$E_{res_{percentage}}\left(\%\right)=\frac{E_{res}}{E_{init}}\times 100$$

**Figure 12:**
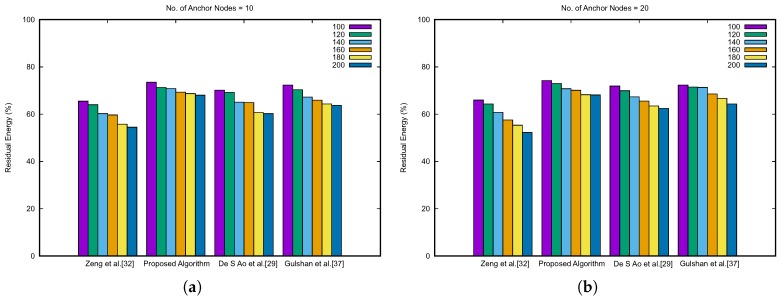

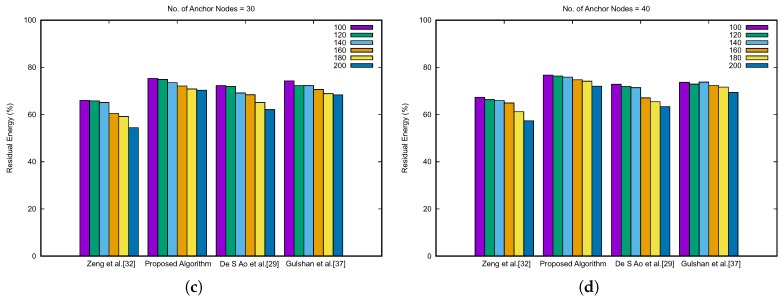
Residual energy comparison.

With the minimum usage of message exchange between anchor nodes and unknown nodes and the handing over the optimization and localization tasks on BS, our proposed approach significantly improves the residual energy of the overall network scenario. Figure 12 describes the result for the network lifetime comparison. The data are represented by varying the anchor nodes from 10 to 40. The data are plotted for a number of unknown nodes vs. the percentage of residual energy. In all the circumstances as shown in Figure 12, the percentage of residual energy for our proposed algorithm is higher than the other algorithms in comparison. Moreover, it shows an effective point for our proposed algorithm: With the increasing number of anchor nodes, the energy residual amount also increases. This signifies that the load is balanced and distributed, for which overall residual energy increases. Furthermore, we have analyzed the complexity of algorithms, which are compared in Table 4, where *m* is the number of anchor nodes used for localization and *n* is the number of unknown nodes.

## 8. Conclusions

In the present study, a novel algorithm is proposed to optimize anchor nodes in CDS. In this case, the algorithm used random mobility for both the anchor as well as unknown nodes and BS was considered to perform the major tasks, such as optimization of CDS, localization with optimized CDS, etc. The vector-based swarm method used for optimization process converges to the global optima rather than the local optima. By keeping anchor nodes and unknown nodes to a minimum working level, traffic overload in the network and message-processing functions are substantially reduced. The localization process is executed with a transformation matrix of nonlinear equations and applying the standard least-square method. The simulated results were compared with recent algorithms. The accuracy and network lifetime are approximately 30% and 67%, respectively, better than the other compared approaches. This algorithm can be used in a dynamic environment, as it converges to the global optimum solution with a lower number of iterations. Additionally, the method is scalable and works with different network sizes, which makes it adaptable to various applications.

## Figures and Tables

**Table 1:** Addressing problem with proposed solution.

Existing Problems	Solution in the Proposed Algorithm
Algorithms are not load-balanced and, therefore, partial exhaustion of the network exists.	Load allocation attribute has been used with $CDS_{\theta }$ and $Al_{\theta }$.
Redundancy of clustering nodes make the process complex and also causes heavy functioning load on some particular nodes.	Redundancy is removed as the CDS is maintained only by anchor nodes having a maximum degree, so that other nodes can be at rest and can be used later.
Validation for overall mobile (sensor and anchor: Both are mobile) environment is not ensured.	In the proposed solution, we have considered all anchor nodes and sensor nodes to be mobile and therefore support full mobility of the network.

**Table 2:** Simulation parameters.

Simulation Area	100 × 100 m${}^{2}$
No. of unknown nodes	100 to 200
No. of anchor nodes	10 to 40
Mobility	Random
Population size	10 to 40
Mutation probability	0.2

**Table 3:** Statistical values of experimentation.

No. of Anchor Nodes	No. of Unknown Nodes	Performance of Proposed Algorithm		
		Average Localization Error	Optimized CDS Size	Comp. Time (s)
10	100	0.0291	5	4.768
10	120	0.0338	5	6.984
10	140	0.0379	6	7.883
10	160	0.0534	7	9.111
10	180	0.0549	7	10.254
10	200	0.0588	7	13.532
20	100	0.0277	8	6.777
20	120	0.0279	8	7.19
20	140	0.0388	10	10.657
20	160	0.0397	10	12.097
20	180	0.0444	11	14.541
20	200	0.0487	11	17.234
30	100	0.0276	11	8.675
30	120	0.0295	12	9.892
30	140	0.0387	13	12.01
30	160	0.0455	13	13.542
30	180	0.0487	14	13.987
30	200	0.0499	14	16.001
40	100	0.0243	16	10.278
40	120	0.0377	16	13.985
40	140	0.0487	16	14.146
40	160	0.0489	20	15.675
40	180	0.0502	20	16.999
40	200	0.0548	22	19.333

**Table 4:** Complexity comparison.

Algorithms	Complexity
De S Ao et al. [29]	O($m^{2}+n$)
Zeng et al. [32]	O($m^{n}+n$)
Gulshan et al. [37]	O($m^{n}+log_{2}n$)
Proposed Algorithm	O($nm\;log_{n}$)
